# Metagenomic and Culture-Based Insights into Salinity-Driven Bacterial Community Dynamics throughout Crude Oil-Degrading Enrichment Cultivation

**DOI:** 10.4014/jmb.2508.08050

**Published:** 2026-04-06

**Authors:** Tuyen Thi Do, Ve Van Le, Loi Thi Thanh Nguyen, Thanh Thi Kim Nguyen, Nguyen Thi Hanh Vu, Hoang Ngoc Trinh, Sang-Ah Lee, Cuong Cao Ngo, Quyet-Tien Phi

**Affiliations:** 1 Graduate University of Science and Technology, Vietnam Academy of Science and Technology, 18 Hoang Quoc Viet Road, Nghia Do, Ha Noi 100000, Vietnam; 2 Institute of Biology, Vietnam Academy of Science and Technology, 18 Hoang Quoc Viet Road, Nghia Do, Ha Noi 100000, Vietnam; 3 Joint Vietnam–Russia Tropical Science and Technology Research Center, 63 Nguyen Van Huyen, Nghia Do, Ha Noi 100000, Vietnam; 4 Faculty of Biotechnology, College of Applied Life Sciences, Jeju National University, 102 Jejudaehak-Ro, Jeju 63243, Republic of Korea; 5 Thai Nguyen University of Sciences, Thai Nguyen City, Thai Nguyen Province 250000, Vietnam

**Keywords:** Crude oil degradation, Hydrocarbon-degrading bacteria, Saline soil, Metagenomics, Bioremediation

## Abstract

Soil salinization and crude oil contamination are critical global threats to ecosystems, agriculture, and human health. Bioremediation is widely recognized as a cost-effective and eco-friendly strategy for removing petroleum pollutants from soil. In this study, we investigated salinity-driven bacterial community dynamics collected from crude oil–contaminated soil in Cam Ranh Bay, Khanh Hoa, over a 21-day enrichment cultivation, using shotgun metagenomic and culture-based approaches. The enrichment cultivation was performed in Bushnell–Haas mineral salts (BHMS) medium supplemented with 5% (v/v) crude oil–diesel mixture (5:95) and 1.5% NaCl. Shotgun metagenomic analysis revealed that after 21 days of enrichment, the relative abundance of crude oil–degrading genera increased markedly in the enriched samples compared to the native samples—for example, *Pseudomonas* rose from 0.44% to 3.51%, *Gordonia* from 0.03% to 78.68%, and *Achromobacter* from 0.03% to 3.77%. Functional analysis further identified metabolic pathways, including hydrocarbon degradation, osmoprotection, and heavy metal detoxification. In addition, 36 representative bacterial strains were isolated from the enriched cultures, predominantly belonging to the genera *Pseudomonas*, *Bacillus*, *Stenotrophomonas*, and *Achromobacter*. All isolates were able to degrade crude oil under salinity stress conditions of up to 4%. Notably, *Rhodococcus* sp. KH5 and *Gordonia* sp. KH53 maintained consistently high degradation efficiencies across 0–4% salinity, ranging from 17.67–35.00% and 28.67–36%, respectively. Overall, our findings demonstrate that saline enrichment shifts the bacterial community toward halotolerant hydrocarbon and crude oil degraders.

## Introduction

As crude oil is a crucial resource for the energy and chemical industries, its demand has been steadily increasing [1]. However, oil spills resulting from accidental releases, transportation activities, and refining processes have caused severe environmental pollution worldwide [2]. Oil contamination can alter the physical and chemical properties of soil, thereby inhibiting plant and microbial life [3]. Since the degradation of crude oil pollutants occurs slowly under natural conditions, developing effective methods for removing oil contaminants from soil has become an important research focus [2].

Several approaches have been developed to remediate crude oil–contaminated soils, including chemical, physical, and biological methods [4]. Each technique has its own advantages and limitations [5]. For example, solvent extraction is an effective approach for remediating oil-contaminated soils, as it offers high removal efficiency, low energy requirements, and rapid pollutant elimination; however, it can also cause secondary pollution due to residual solvents [5]. Microorganisms play a vital role in soil biological activity, contributing to the decomposition, conversion, and other biochemical processes of organic matter [6]. Bioremediation is widely employed as an environmentally friendly approach to eliminate petroleum pollutants from soil [7]. Compared with costly physical equipment and chemical reagents, microorganisms provide a cost-effective alternative by mineralizing hydrocarbons to carbon dioxide through intracellular and extracellular enzymes [8]. Owing to their widespread distribution, ease of cultivation, and diverse metabolic pathways, bacteria represent the most active and crucial group among oil-degrading microorganisms [9].

Only a small fraction of natural microbial communities can metabolize hydrocarbons in crude oil-contaminated environments [10, 11]. During enrichment cultivation, selective pressure from crude oil and nutrients favors the growth of microbes with the capacity for hydrocarbon degradation and biosurfactant production [10]. Therefore, this approach enables the recovery of dominant oil-degrading strains or consortia, which can subsequently be isolated, characterized, and harnessed for bioremediation applications [12].

Soil salinization has emerged as one of the major environmental and socio-economic challenges worldwide [13, 14]. Oil-contaminated sites are frequently exposed to saline stress conditions [15, 16]. These overlapping stressors—salinity and hydrocarbon pollution—pose serious environmental challenges [16-18]. In Vietnam, soil salinization continues to expand as a consequence of climate change and rising sea levels [19]. Among the affected areas, Cam Ranh Bay in Khanh Hoa province is particularly concerning, as its soils are heavily contaminated with diesel, with total petroleum hydrocarbon concentrations of up to 4.052 mg/kg [20]. Therefore, elucidating the role of microorganisms in saline, oil-contaminated soils is crucial not only for advancing our understanding of microbial ecology under multiple stress conditions but also for developing effective strategies for bioremediation and sustainable soil management in Vietnam.

Taken together, this study aimed to: (1) investigate the structure and diversity of bacterial communities in saline, oil-contaminated soil; (2) provide insights into salinity-driven bacterial community dynamics during crude oil-degrading enrichment cultivation; and (3) isolate and evaluate the degradation capacity of bacterial strains obtained from enrichment cultures in saline conditions. Our results demonstrate that enrichment cultivation under saline conditions favored the enrichment of hydrocarbon-degrading bacteria with salinity tolerance and potential plant growth–promoting capabilities. The isolation of strains with high degradation capacities further underscores their potential as effective candidates for the bioremediation of crude oil in saline soils.

## Materials and Methods

### Soil Sampling and Physicochemical Characterization

Soil samples were collected in triplicate (designated as N1, N2, and N3) from Cam Ranh Bay, Khanh Hoa Province, Vietnam (GPS: 12°02'41"N 109°11'39"E) using a sharp-edged steel cylinder manually driven into the soil [21]. Given the long-standing history of crude oil contamination at the sampling site, oil has infiltrated the deeper soil layers. Accordingly, soil samples were collected from a depth of 0-60 cm, which was also selected based on a previous study [22]. The samples were sieved through a 2 mm mesh to determine soil physicochemical properties and immediately transported to the laboratory in polyethylene bags at 4°C.

Soil moisture content was determined based on weight loss after oven-drying at 105°C to a constant weight. Soil pH was measured in 1 M KCl solution using a pH meter (Hanna HI2002-02, TB.H.70). Electrical conductivity (EC) was determined using a conductivity meter (Hanna HI98192, TB.H.44). Total soluble salt content was measured by extraction followed by electrical conductivity determination in a saturated extract using a conductivity meter. Cation exchange capacity (CEC) was measured by cation exchange with ammonium acetate solution using a nitrogen distillation unit, a cation exchange column, and an analytical balance [23]. Organic carbon content was determined by oxidizing soil organic matter with potassium dichromate in a sulfuric acid medium, followed by titration of the remaining dichromate. The organic carbon content was calculated based on the amount of dichromate consumed in the reaction [24]. Nitrogen in the soil sample was converted to ammonia through digestion with H_2_SO_4_ in the presence of a catalyst. The released ammonia was then absorbed into a receiving solution and quantified by titration. Total phosphorus was quantified using atomic absorption spectroscopy and atomic emission spectroscopy with a UV-VIS spectrophotometer (Jasco V-730). Soil particle fractions were separated using a mixture of sodium hexametaphosphate and sodium carbonate. Potassium concentration was measured by flame photometry or emission spectroscopy after sample digestion with hydrofluoric and perchloric acids. Total sulfur was determined by converting sulfur-containing compounds to soluble sulfate (SO_4_^2-^) and quantifying sulfate content gravimetrically. Total petroleum hydrocarbons (TPHs) were determined according to the Standard Methods for the Examination of Water and Wastewater [25].

### Enrichment of Crude Oil-Degrading Bacterial Communities

For enrichment cultivation, 5 g of soil was inoculated into 500 ml Bushnell-Haas mineral salts medium (BHMS) supplemented with 5% (v/v) crude oil-diesel mixture (5:95) and 1.5% NaCl, and incubated for 21 days at 28°C with shaking at 180 rpm [26]. At 7-day intervals, 40 ml of enrichment culture was collected: 30 ml was centrifuged at 12,000 rpm for 20 min at 4°C and the pellet was stored at -80°C for shotgun metagenomic analysis, while 10 ml was stored at 4°C for bacterial isolation [27]. As a control, BHMS containing 5% (v/v) crude oil-diesel mixture (5:95, v/v) without soil inoculum was incubated under identical conditions to assess abiotic losses of crude oil.

### Isolation and Identification of Crude Oil-Degrading Bacterial Strains

For bacteria isolation, 100 μl of enriched culture was serially diluted, and 100 μl aliquots of 10^-4^ to 10^-6^ dilutions were spread onto Bushnell–Haas agar (Himedia, India) supplemented with 1.5% NaCl and 1% crude oil. The plates were incubated at 30°C for 2 days, and 36 morphologically distinct colonies were purified by repeated streaking on Nutrient Agar (Himedia).

For bacterial identification, genomic DNA was extracted using the ZR Fungal/Bacterial DNA MiniPrep kit (Zymo Research, USA) following the manufacturer’s instructions. The 16S rRNA gene was amplified and sequenced with the universal primer set 27F (5′-AGAGTTTGATCATGGCTCAG-3′) and 1492R (5′-TACGGYTACCTTGTTACGACTT-3′) [28]. The obtained sequence was compared against all validly published species in the EzBioCloud database (accessed on 21 April, 2025) [29]. The 16S rRNA gene sequences of the isolated strains and those of the nearest phylogenetic type strains retrieved from EzBioCloud were aligned using MUSCLE with default parameters [30]. Phylogenetic trees were constructed using the MEGA version 11 [31], using the neighbor-joining algorithm [32] with 1000 bootstrap replications. Evolutionary distances were calculated using Kimura’s two-parameter model [33].

### Crude Oil Degradation Efficiency

The residual crude oil in the enrichment culture broth was determined gravimetrically according to Standard Methods for the Examination of Water and Wastewater (SMEWW) 5520B&F: 2023. Residual oil was extracted with n-hexane, and the solvent was evaporated to constant weight before measuring the oil mass. A control flask containing mineral medium and oil without bacterial inoculum was incubated under identical conditions to assess abiotic losses.

The degradation efficiency was calculated according to the formula:

Degradation (%) = 100 × (C_i_ − C_f_) / C_i_

where C_i_ and C_f_ denote the initial and final oil concentrations (g), respectively.

### Screening of Bacterial Strains for Crude Oil Biodegradation Capability

Bacterial strains were inoculated into Bushnell–Haas broth supplemented with 0–4% NaCl, 0.5% (w/v) 2,6-dichlorophenol indophenol (2,6-DCPIP), 0.1% Tween 80, and 1% (v/v) crude oil, and incubated at 28°C with shaking at 180 rpm for 24 h [34]. Color change from blue to colorless was monitored daily. Control experiments were prepared without inoculum, and all treatments were conducted in triplicate. After 7 days of incubation at 28°C, the medium was filtered to remove biomass, and the filtrate was centrifuged at 8000 rpm for 15 min. The absorbance of the supernatant was measured at 609 nm to quantify residual DCPIP. Percent degradation was calculated as [34]:

Degradation (%) = 100 × [1 − (absorbance of treated sample/absorbance of control)]

### Shotgun Metagenomic Sequencing and Data Processing

DNA was extracted using the DNeasy PowerSoil Pro Kit (Qiagen, USA). Shotgun sequencing libraries were prepared using the NEBNext dsDNA Fragmentase and NEBNext Ultra II DNA Library Prep Kit for Illumina (NEB, USA) following the manufacturer’s instructions and sequenced on an Illumina NovaSeq platform with 150 bp paired-end reads. Raw sequencing data were processed using fastp to remove adapters and low-quality reads. Potential host-derived sequences were removed using Kraken2 [35]. The resulting high-quality reads were taxonomically classified with Kraken2 against a database comprising bacteria, archaea, protozoa, fungi, and viruses. Relative taxonomic abundance was calculated based on the proportion of classified reads assigned to each taxon relative to the total number of classified reads per sample.

For functional analysis, the reads were *de novo* assembled with metaSPAdes version 3.15.4 [36]. Prodigal version 2.6.3 was used to predict coding DNA sequences (CDSs) from the assembled contigs. EggNOG-mapper2 [37] with the EggNOG-v5 database [38] was employed to annotate the predicted CDSs. The assigned functions were then used to infer the metabolic pathways with MinPath based on the MetaCyc and KEGG databases with default parameters.

Functional pathway abundance was quantified from metagenomic sequencing data using TPM (transcripts per million) normalization. This normalization enables comparison of relative pathway abundance across samples [39, 40]. Specifically, pathways involved in hydrocarbon degradation and associated metabolic processes were selected prior to statistical analysis. For differential analysis, TPM values were log_2_-transformed after addition of a pseudocount of 0.5 to all samples. The pseudocount was introduced to avoid undefined values arising from zero or near-zero baseline abundance and to reduce inflation of fold-change estimates. Log_2_ fold-change (log_2_FC) between time points was calculated as the difference in mean log_2_-transformed TPM values:

log_2_FC*_DayX_* = mean (log_2_ (TPM + 0.5))*_DayX_* − mean (log_2_ (TPM + 0.5))*_Day7_*

where Day 7 served as the reference condition.

Statistical significance between groups was assessed using two-sided Student’s *t*-tests performed on log_2_-transformed abundance values. Resulting *p*-values were adjusted for multiple testing using the Benjamini-Hochberg method, and adjusted *p*-values < 0.05 were considered statistically significant.

### Statistical Analysis

Analyses were conducted in R version 4.4.1. The *microbiotaProcess* package (version 1.19.0) was used to calculate alpha diversity indices [41]. Principal component analysis (PCA) was performed and visualized using the ggfortify package [42] in combination with ggplot2 [43]. Differences in bacterial species composition between native and enrichment cultivations were assessed using the *indicspecies* package (version 1.8.0) [44]. An indicator species was considered a valid indicator when its indicator value exceeded 0.6 and the associated *p*-value was < 0.05, as determined by 999 permutation tests. The tidyverse package (version 2.0.0) was used to generate horizontal bar plots showing log2 fold-changes of pathway abundances at days 14 and 21 relative to the day 7 baseline during soil enrichment [45]. Heatmaps with hierarchical clustering were produced using the pheatmap package (version 1.0.12) to classify bacterial isolates according to their oil-degradation performance under increasing salinity [46]. Statistical analysis was performed using GraphPad Prism version 10.2.3 (GraphPad Software, Inc., USA). All experiments were conducted in triplicate, and data were expressed as mean ± standard deviation. Statistical significance (*p* < 0.05) was evaluated by one-way analysis of variance (ANOVA) followed by Tukey’s multiple comparison test.

## Results

### Physicochemical Properties and Petroleum Hydrocarbon Content of Soil Samples

The physicochemical properties and concentrations of TPHs in three native soil samples (N1, N2, and N3) are summarized in Table 1. Moisture content, porosity, and particle-size fractions (sand, silt, clay) were similar among samples. Soil pH ranged from 6.87 to 7.09, indicating near-neutral conditions. The total organic matter content of N1 was 1.69%, higher than that of N2 (1.62%) and N3 (1.56%). Total dissolved salts and EC ranged from 0.293 to 0.297% and 2.127 to 2.150 dS m^-1^, respectively. TPH concentrations in all three soil samples ranged from 3,823 to 3,854 mg kg^−1^ (Table 1), which exceeded both the reported background level of 1,965 ± 16 [47] and the Vietnamese soil quality standard (≤500–2,000 mg kg^−1^; QCVN 03:2023/BTNMT).

### Crude Oil Degradation Efficiency in Enrichment Cultures

**Fig. 1:**
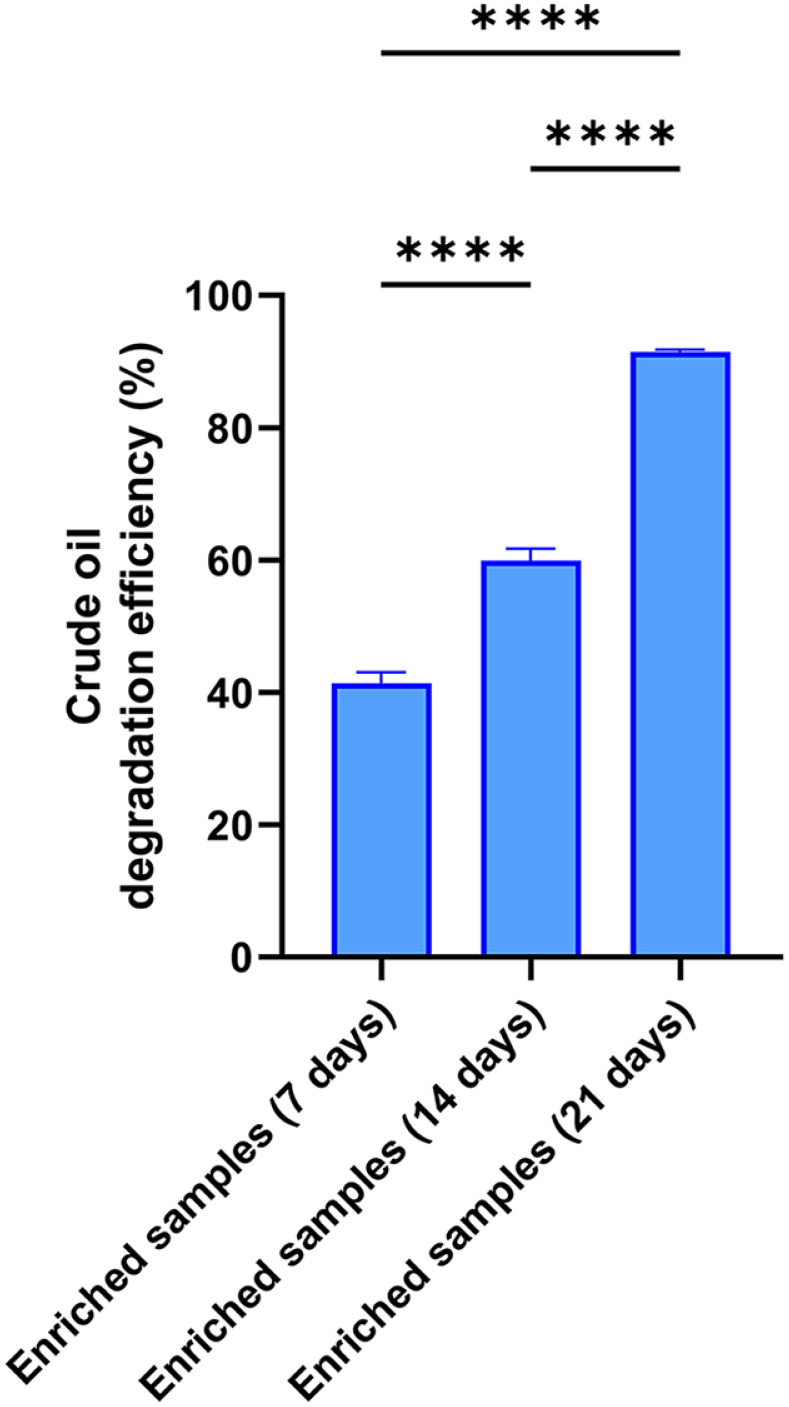
Crude oil degradation efficiency of the bacterial community. **** indicates significant differences (*p* < 0.0001). Error bars indicate standard deviation of three replicates.

To assess whether selective enrichment favored hydrocarbon-degrading microorganisms under saline conditions, the crude oil degradation efficiency of bacterial communities was measured on days 7, 14, and 21 to evaluate the metabolic activity of the consortium. Crude oil removal by the enriched consortium increased progressively over the cultivation period (Fig. 1). The bacterial community degraded 41.38% of crude oil after 7 days of enrichment (ER7), 60.82% after 14 days of enrichment (ER14), and 91.00% after 21 days of enrichment (ER21).

### Bacterial Community Composition and Diversity

**Fig. 2:**
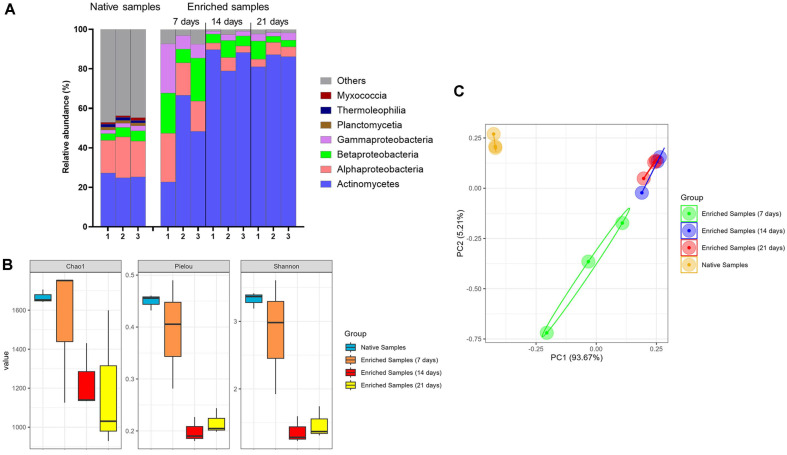
Bacterial community composition at the class level (A) alpha diversity indices (B) and beta diversity (C) in native and enriched samples.

To elucidate the dynamics of bacterial community composition, enrichment cultures were collected at 7-day intervals over a 21-day period. The proportion of classified reads was lower in the native soil samples than in the enrichment samples, indicating that native soils harbor a high proportion of uncharacterized taxa that are not represented in current taxonomic reference databases (Table S1). The bacterial community of the crude oil-contaminated saline soil was dominated by the phyla Actinomycetota and Pseudomonadota (Fig. S1). At the class level, bacterial communities were dominated by Actinomycetes (22.73-89.71%), Alphaproteobacteria (3.31-24.68%), Betaproteobacteria (3.04-21.83%), and Gammaproteobacteria (1.54-24.87%) (Fig. 2A). The abundance of Actinomycetes increased markedly from 25.80 ± 1.25% in native samples to 84.78 ± 3.26% in the enrichment culture on day 21, while that of Pseudomonadota declined from 8.62 ± 0.13% to 0.46 ± 0.39% over the same period. The richness and diversity of the native samples and enriched samples at day 7 were higher than those observed in the enriched samples at days 14 and 21 (Fig. 2B). PCA revealed that the bacterial community formed distinct clusters according to group, indicating that the composition of the bacterial community changed after enrichment (Fig. 2C). The bacterial community composition did not differ significantly between day 14 and day 21 of enrichment (PERMANOVA *p* = 0.8).

### Bacterial Community Dynamics and Functional Features during Enrichment Cultivation

**Fig. 3:**
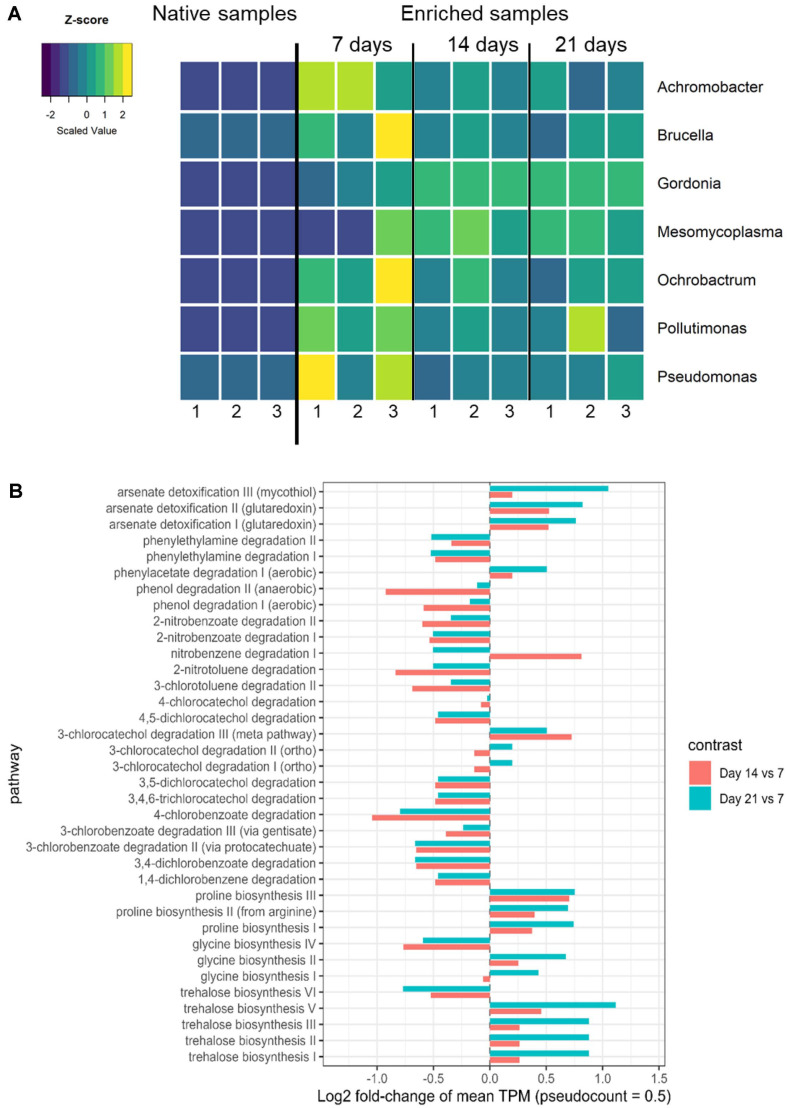
Temporal dynamics of the bacterial community during the enrichment process (**A**) and differential changes in metabolic pathway abundances (log2 fold-change) at Days 14 and 21 relative to the Day 7 baseline (**B**). Bars represent mean log2 fold-change values calculated from TPM-normalized pathway abundances.

To identify the genera enriched in the cultivation compared with the native sample, we analyzed indicator species. Indicator species analysis revealed that the genera *Achromobacter*, *Brucella*, *Gordonia*, *Mesomycoplasma*, *Ochrobactrum*, *Pollutimonas*, and *Pseudomonas* were more abundant in the enriched samples than in the native samples (Fig. 3A). Functional prediction identified multiple metagenome-derived metabolic pathways directly related to the degradation of petroleum hydrocarbons in the enriched bacterial communities, including phenol degradation I–II, phenylacetate degradation I, and phenylethylamine degradation I–II, which represent central routes for the breakdown of monoaromatic and related hydrocarbon compounds (Fig. 3B). In addition, the communities harbored pathways associated with salinity resistance, such as proline biosynthesis I–II, trehalose biosynthesis I–VI, and glycine biosynthesis I–IV, indicating the capacity to adapt to osmotic stress in oil-contaminated soils. Beyond hydrocarbon degradation and stress adaptation, several xenobiotic-related pathways were also detected, notably those involved in chlorinated and nitroaromatic compounds, including 1,4-dichlorobenzene degradation, 3,4-dichlorobenzoate degradation, 3-chlorobenzoate degradation I/III, 4-chlorobenzoate degradation, nitrobenzene degradation I, and 2-nitrobenzoate degradation I–II. Additionally, pathways associated with heavy metal and cyanide detoxification (*e.g.*, arsenate detoxification I–III and cyanide detoxification I) were identified, indicating adaptive capacities of the enriched microbial communities (Fig. 3B).

### Identification and Degradation Efficiency of Crude Oil-Degrading Bacterial Strains

**Fig. 4:**
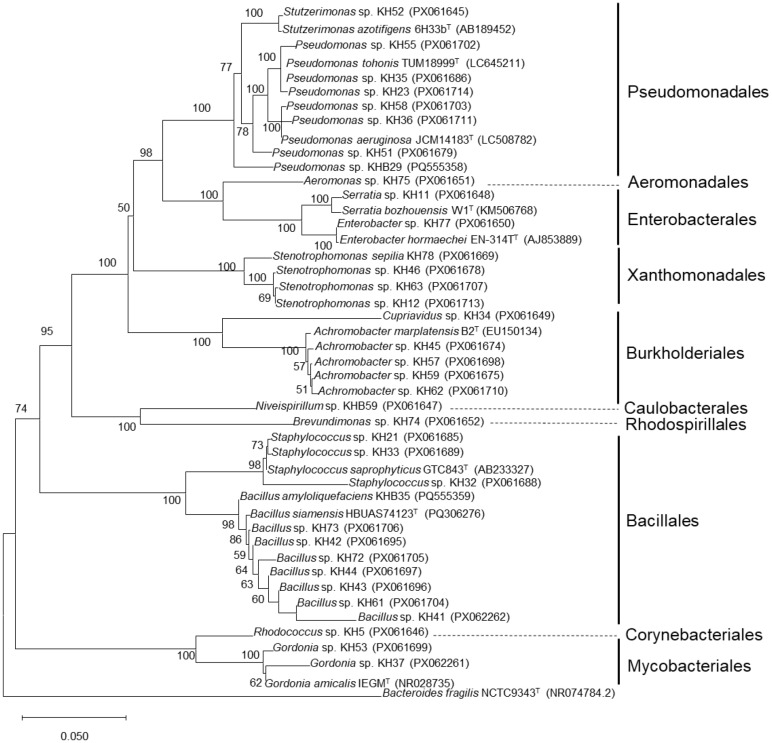
Neighbor-joining phylogenetic tree based on 16S rRNA gene sequences of petroleum hydrocarbon-degrading strains isolated from enriched samples. Values in parentheses show GenBank accession number of the respective strains. Scale bar, 0.05 nucleotide substitutions per nucleotide position.

**Fig. 5:**
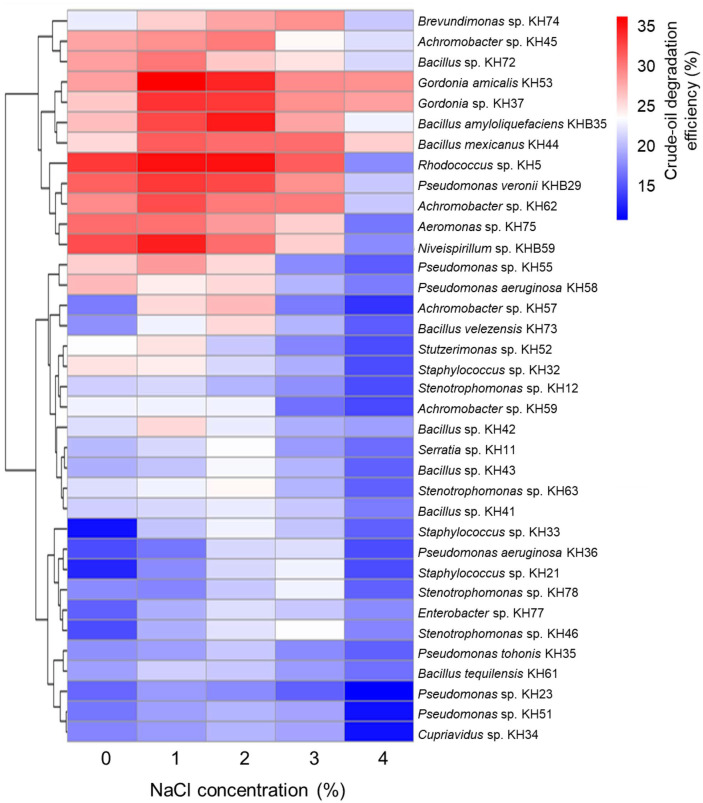
Heatmap with hierarchical clustering of bacterial isolates based on crude-oil degradation efficiency measured at different NaCl concentrations (0, 1, 2, 3, and 4%). Color scale represents crude-oil degradation percentage.

A total of 36 strains were isolated from the enriched samples, spanning diverse 9 orders (Pseudomonadales, Aeromonadales, Enterobacterales, Xanthomonadales, Burkholderiales, Caulobacterales, Rhodospirillales, Bacillales, and Mycobacteriales) (Fig. 4). At the genus level, they were predominantly assigned to *Pseudomonas* and *Achromobacter* (Table S2). All strains were capable of degrading crude oil (Fig. 5), with most exhibiting optimal activity at 1–2% NaCl and reduced performance efficiency at concentrations ≥3% (Fig. 5 and Table S3). Notably, *Rhodococcus* sp. KH5 and *Gordonia* sp. KH53 exhibited high crude oil degradation capabilities across a broad salinity range. Strain KH5 degraded 33.00%, 35.00%, 31.33%, and 17.67% of crude oil at 0%, 1–2%, 3%, and 4% NaCl, respectively, while KH53 achieved 28.00% at 0%, 36.00% at 1%, 34.00% at 2%, 29.00% at 3%, and 28.67% at 4% (Fig. 5 and Table S3). In addition, *Niveispirillum* demonstrated crude oil-degrading activity for the first time, with the highest degradation rate (34.33%) observed at 1% NaCl.

## Discussion

Soil salinization and crude oil contamination are global threats, particularly in coastal oil-producing regions where rising sea levels intensify saline stress. Such conditions hinder plant and microbial growth, lower hydrocarbon and oxygen solubility, and reduce soil porosity [48]. Bioremediation offers a cost-effective and environmentally sustainable strategy to address these challenges compared to conventional physicochemical approaches. Since only a small fraction of microbes are capable of degrading crude oil, enrichment cultivation is essential to selectively enhance crude oil-degrading bacteria prior to isolation [10]. Therefore, we investigated the dynamics of bacterial communities in saline, oil-contaminated soils during enrichment cultivation using shotgun metagenomic and culture-based approaches, and assessed the degradation capacities of isolated strains to evaluate their potential for bioremediation in saline oil-contaminated environments.

To ensure relevance to saline oil-contaminated conditions, the soil samples used in this study were collected from a coastal area. EC can be used as an indicator of soil salt content [49]. According to the USDA classification, soils with an ECe of 0–2 dS m^-1^ are considered non-saline, whereas those with an ECe of 2–4 dS m^-1^ are classified as slightly saline [50]. The soils analyzed in this study fell within the slightly saline range, with EC values of 2.127–2.150 dS m^-1^, a level that can negatively affect crop productivity and soil microbiota [51]. Since TPH concentration indicates the extent of oil contamination, it was measured to evaluate soil pollution levels [52]. TPH concentration of the native samples ranged from 3823.25 to 3854.93 mg kg^-1^, exceeding the Dutch Intervention Value (IV) thresholds of over 500 mg kg^-1^ for TPH in soils, a benchmark widely applied across European countries [52, 53]. These levels indicate severe oil contamination, necessitating remediation to mitigate risks to ecosystems, agriculture, and human health [54].

Enrichment cultivation is known to reduce microbial alpha diversity [10]. Consistently, the enrichment culture samples on days 14 and 21 exhibited lower richness and diversity than the native soil samples (Fig. 2B). This reduction likely resulted from the selective pressure of the enrichment conditions, which favored bacteria with specific metabolic capabilities and adaptability (Fig. 2B). The addition of nutrients and crude oil alters the composition of the bacterial community [10]. As anticipated, the community structure during enrichment cultivation differed markedly from that of the native samples, with crude oil–degrading genera being more abundant (Figs. 2A and 3A). The enrichment of *Achromobacter*, *Gordonia*, and *Pseudomonas* during cultivation, as revealed by shotgun metagenomic analysis, was further corroborated by culture-based methods (Figs. 3A and 4). The distinct bacterial assemblages observed between ER7 and ER14/ER21 indicated clear temporal shifts in community composition during enrichment (Fig. 3A). Early-stage communities (ER7) were dominated by *Achromobacter*, likely due to its rapid growth and metabolic versatility [55]. As an important genus in crude oil degradation, *Achromobacter* has been frequently detected in crude oil-polluted saline sites [56, 57] and can tolerate high NaCl concentration [58, 59]. By contrast, at later stages (days 14 and 21), *Gordonia* became dominant, in line with its known halotolerance and hydrocarbon-degrading capabilities [60]. The increased abundance of *Gordonia* species during enrichment cultivation in this study is consistent with previous reports [61]. They can degrade dibenzothiophene, a major polycyclic aromatic sulfur heterocyclic in crude oils [62]. *Gordonia iterans*, isolated from crude oil-contaminated marine sediments, has been shown to remove up to 84.2% of crude oil in Bushnell–Haas medium [63]. The ability of *Pseudomonas* to degrade crude oil in saline conditions has been widely known [9, 64]. *Pseudomonas* species produce alkane hydroxylase enzymes that enable the oxidation and subsequent metabolism of alkane compounds [65]. Microbial growth in natural environments often depends on strict or unknown nutritional and physiological requirements, which makes many species difficult to culture under laboratory conditions [66]. Therefore, fewer than 1% of bacterial species can be successfully cultivated in the laboratory [67]. In this study, some bacterial taxa, such as *Brucella* and *Pollutimonas*, detected by shotgun metagenomics were not recovered through cultivation, likely due to their fastidious growth requirements. The genus *Pollutimonas* was recently described by Babich *et al*. and has been reported in groundwater, activated sludge, seawater, and soil [68]. This study is the first to report the presence of *Pollutimonas* in saline, crude oil-contaminated soil, highlighting its potential ecological role under combined salinity and hydrocarbon stress.

To elucidate how bacterial communities can adapt under enrichment cultivation, we analyzed their functional features. The functional prediction revealed pathways that are directly linked to the degradation of TPHs, which encompass a wide spectrum of aliphatic and aromatic hydrocarbons and their derivatives [69, 70] (Fig. 3B). Specifically, pathways such as phenol degradation I, phenol degradation II, phenylacetate degradation I, phenylethylamine degradation I–II, nitrobenzene degradation I, and 2-nitrobenzoate degradation I–II were detected (Fig. 3B). These routes converge on central catabolic pathways, particularly catechol and protocatechuate metabolism, ultimately leading to the mineralization of hydrocarbons into CO_2_, H_2_O, and biomass [71, 72]. The presence of such diverse aromatic degradation capacities strongly suggests a functional potential of the enriched communities for reducing TPH levels in contaminated soils [69, 70]. In addition, xenobiotic-related pathways, including 1,4-dichlorobenzene degradation, 3,4-dichlorobenzoate degradation, and 3-chlorobenzoate degradation I/III, highlight the metabolic versatility of these communities in processing structurally diverse hydrocarbons and xenobiotic derivatives, which often co-occur along with petroleum hydrocarbons in oil-contaminated soils.

Beyond hydrocarbon degradation, the enriched communities also carried pathways related to environmental adaptation. High salinity is a common stressor in oil-polluted soils, as it disrupts microbial osmotic balance and reduces metabolic activity [73]. The detection of proline biosynthesis I–II, trehalose biosynthesis I–VI, and glycine biosynthesis I–IV—all encoding well-known osmoprotectants [74, 75]—suggests that the communities possess mechanisms for osmoprotection and survival under salt stress. The enrichment of plant growth-promoting (PGP) genera such as *Achromobacter* [76], *Gordonia* [77], *Pseudomonas* [78], and *Ochrobactrum* [79], together with the detection of auxin biosynthesis pathways (indole-3-acetate biosynthesis I–V), further indicates the potential of these communities to contribute to rhizosphere resilience and plant growth under saline oil-stress conditions at soil-sampling sites. Moreover, considering that oil-contaminated soils often contain both hydrocarbons and heavy metals [80], the detection of detoxification pathways such as arsenate detoxification I–III and mercury detoxification underscores additional adaptive traits that may enhance bioremediation performance in complex environments. Although certain pathways exhibited trends toward increased abundance at Day 14 or Day 21, these differences did not reach statistical significance (*p* ≥ 0.05). This suggests that longer enrichment time may contribute to gradual enhancement of crude-oil degradation–related pathways. However, further investigation with extended time points or increased replication would be required to confirm this effect.

To identify superior crude oil-degrading bacteria, we evaluated the degradation efficiency of strains isolated from the enriched sample. Notably, *Rhodococcus* sp. KH5 and *Gordonia amicalis* KH53 maintained high crude oil degradation activity across a broad salinity range (Fig. 5). These results highlight their potential as promising candidates for bioremediation of crude oil in saline soils. Members of the genus *Rhodococcus* are well known for their crude oil-degrading capabilities [81-83]. This genus possesses a broad repertoire of genes associated not only with hydrocarbon degradation but also with plant growth promotion and tolerance to diverse environmental stresses [84]. Because microorganisms require diverse enzymes for crude oil metabolism and degradation, a single species is insufficient to achieve complete degradation [85]. Therefore, further research is needed to construct microbial consortia from isolated strains that can act synergistically to enhance hydrocarbon degradation.

The genus *Niveispirillum* was first described by Lin *et al*. [86]. Although *Niveispirillum* species have been reported to exhibit nitrogen-fixing [87] and lignolytic activity [88], their ability to degrade crude oil has not previously been documented. To our knowledge, this is the first report of the crude-oil degradation capacity of a strain of *Niveispirillum*. The crude oil degradation capacity of strain KHB59, identified in this study, may provide new perspectives on the use of the genus *Niveispirillum* in oil-contaminated soil remediation. Lipases play a crucial role in crude oil degradation [89]. As *Niveispirillum* species are capable of producing lipases [86], we hypothesize that lipase activity may contribute to their hydrocarbon-degrading potential. Further studies are required to elucidate the metabolic mechanisms underlying crude oil degradation by this genus.

In conclusion, our study demonstrates that enrichment cultivation shaped microbial communities with strong functional capacities for the degradation of total petroleum hydrocarbons, while also equipping them with adaptive traits for salinity tolerance, heavy metal detoxification, and plant growth promotion. Such multifunctionality highlights their ecological robustness and potential utility in the bioremediation of oil-contaminated soils. The isolation of bacterial strains with high degradation capacities highlighted their potential as effective candidates for bioremediation of crude oil in saline soils.

## Supplemental Materials

Supplementary data for this paper are available on-line only at http://jmb.or.kr.



## Figures and Tables

**Table 1:** Physicochemical properties and petroleum hydrocarbon contents of soil samples.

Properties	Native samples		
	N1	N2	N3
Physical properties			
Moisture (%)	17.9	17.67	17.8
Porosity (%)	42.3	41.57	41.94
Mechanical composition (%)			
- Sand	90.86	90.84	90.85
- Silt	4.8	5.0	4.9
- Clay	4.34	4.16	4.25
Chemical properties			
pH (pHKCl)	6.87	7.09	6.98
Total soluble salts (%)	0.29	0.30	0.30
Electrical conductivity (EC. dS/m)	2.13	2.15	2.14
Soil absorption capacity (CEC. meq/100g)	2.12	1.81	1.97
Total organic matter (%)	1.69	1.62	1.56
Total nitrogen (%)	0.03	0.08	0.06
Total phosphorus (%)	0.03	0.05	0.04
Total sulfur (%)	0.04	0.04	0.04
Total potassium (%)	0.20	0.22	0.21
Petroleum pollutants			
Total petroleum hydrocarbons (TPH, mg/kg)	3846.76	3854.93	3823.25
