# Supplementary material for: Metagenomic and Culture-Based Insights into Salinity-Driven Bacterial Community Dynamics throughout Crude Oil-Degrading Enrichment Cultivation
Source: J Microbiol Biotechnol. 2026 Apr 6;36:e2508050. doi: 10.4014/jmb.2508.08050 (PMC13062504; doi:10.4014/jmb.2508.08050)
Supplement: Supplementary file 1 [file jmb-36-e2508050-supple.pdf]

J. Microbiol. Biotechnol. 2026. 36: e2508050
https://doi.org/10.4014/jmb.2508.08050

Supplementary Figure and Tables

Metagenomic and culture-based insights into salinity-driven bacterial community dynamics throughout crude oil-degrading enrichment cultivation

Tuyen Thi Do[1,2,3], Ve Van Le[4], Loi Thi Thanh Nguyen[2], Thanh Thi Kim Nguyen[3], Nguyen Thi Hanh Vu[2], Hoang Ngoc Trinh[5], Sang-Ah Lee[4], Cuong Cao Ngo[3], and Quyet-Tien Phi[1,2*]

[1]Graduate University of Science and Technology, Vietnam Academy of Science and Technology, 18 Hoang Quoc Viet Road, Nghia Do, Ha Noi 100000, Vietnam
[2]Institute of Biology, Vietnam Academy of Science and Technology, 18 Hoang Quoc Viet Road, Nghia Do, Ha Noi 100000, Vietnam
[3]Joint Vietnam–Russia Tropical Science and Technology Research Center, 63 Nguyen Van Huyen, Nghia Do, Ha Noi 100000, Vietnam
[4]Faculty of Biotechnology, College of Applied Life Sciences, Jeju National University, 102 Jejudaehak-Ro, Jeju 63243, Republic of Korea
[5]Thai Nguyen University of Sciences, Thai Nguyen City, Thai Nguyen Province 250000, Vietnam

* Correspondence to: tienpq@ib.ac.vn/tienpq@ibt.ac.vn

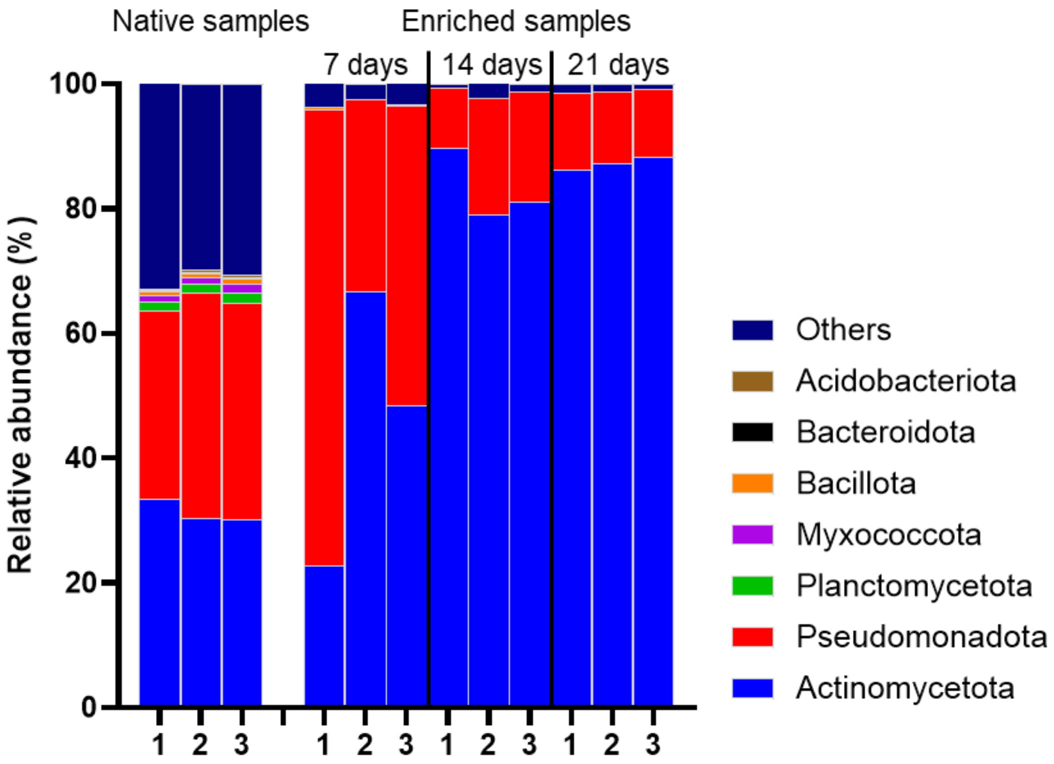

Fig. S1. Bacterial community composition at the phylum level.

Table S1. Summary of metagenomic sequencing and read-processing statistics.

Sample	Total reads	Total bases (bp)	Q30 (%)	Reads after quality filtering	Classified reads (%)	Unclassified reads (%)
N1	21,188,805	6,356,641,500	95.5	21,048,400	17.30	82.7
N2	21,574,302	6,472,290,600	95.4	21,427,097	17.16	82.84
N3	20,024,486	6,007,345,800	95.5	19,876,191	17.90	82.10
ER7_1	16,290,835	4,887,250,500	90.5	13,880,691	81.77	18.23
ER7_2	18,748,268	5,624,480,400	91.7	16,585,018	96.49	3.51
ER7_3	15,628,343	4,688,502,900	90.7	13,142,488	82.24	17.76
ER14_1	24,952,939	7,485,881,700	91.5	21,979,954	98.13	1.87
ER14_2	20,055,854	6,016,756,200	92.0	17,851,230	96.98	3.02
ER14_3	24,793,510	7,438,053,000	92.2	21,852,639	98.6	1.40
ER21_1	18,580,816	5,574,244,800	91.6	16,337,834	97.59	2.41
ER21_2	20,626,572	6,187,971,600	91.3	18,133,147	98.24	1.76
ER21_3	26,311,759	7,893,527,700	92.0	23,238,725	94.42	5.58

Note: Percent classified/unclassified refers to the proportion of quality-filtered reads assigned/unassigned by Kraken2

Table S2. Pairwise 16S rRNA gene sequence similarity between the isolates and their closest type strains based on the EzBioCloud database (accessed on April 21, 2025).

Isolate	Closest type strains	Accession number	Sequence similarity (%)
KH52	*Stutzerimonas azotifigens* 6H33b^T	AB189452	99.02
KH55	*Pseudomonas tohonis* TUM18999^T	LC645211	97.99
KHB35	*Bacillus siamensis* KCTC 13613^T	AJVF01000043	99.92
KH23	*Pseudomonas solani* Sm006^T	LC744517	98.95
KH58	*Pseudomonas aeruginosa* JCM 5962^T	BAMA01000316	98.96
KH36	*Pseudomonas aeruginosa* JCM 5962^T	BAMA01000316	98.27
KH51	*Pseudomonas furukawaii* KF707^T	AJMR01000229	97.98
KHB29	*Pseudomonas violetae* TNT11^T	JAKNRV010000349	99.86
KH75	*Aeromonas hydrophila* subsp. *hydrophila* ATCC 7966^T	CP000462	99.57
KH11	*Serratia bozhouensis* W1^T	KM506768	99.27
KH77	*Enterobacter hormaechei* subsp. *oharae* DSM 16687^T	CP017180	99.23
KH78	*Stenotrophomonas sepilia* SM-16975^T	LXXZ01000044	99.15
KH46	*Stenotrophomonas acidaminiphila* JCM 13310^T	LDJO01000053	99.23
KH63	*Stenotrophomonas acidaminiphila* JCM 13310^T	LDJO01000053	99.44
KH12	*Stenotrophomonas acidaminiphila* JCM 13310^T	LDJO01000053	99.31
KH34	*Cupriavidus neocaledonicus* STM6070^T	FN908230	96.91

KH45	*Achromobacter marplatensis* B2^{T}	EU150134	98.69
KH57	*Achromobacter ruhlandii* ATCC 15749^{T}	AB010840	99.31
KH59	*Achromobacter marplatensis* B2^{T}	EU150134	99.27
KH62	*Achromobacter marplatensis* B2^{T}	EU150134	99.20
KHB59	*Niveispirillum cyanobacteriorum* TH16^{T}	KJ862840	97.01
KH74	*Brevundimonas faecalis* CS20.3^{T}	FR775448	99.09
KH21	*Staphylococcus edaphicus* P5085^{T}	KY315825	99.37
KH33	*Staphylococcus saprophyticus* subsp. *bovis* GTC 843^{T}	AB233327	99.24
KH32	*Staphylococcus caledonicus* H8/1^{T}	MT671558	96.20
KH35	*Pseudomonas tohonis* TUM18999^{T}	LC645211	99.24
KH73	*Bacillus mexicanus* FSQ1^{T}	JAHAWP010000006	99.14
KH42	*Bacillus mexicanus* FSQ1^{T}	JAHAWP010000006	99.45
KH72	*Bacillus tequilensis* KCTC 13622^{T}	AYTO01000043	98.20
KH44	*Bacillus mexicanus* FSQ1^{T}	JAHAWP010000006	98.92
KH43	*Bacillus mexicanus* FSQ1^{T}	JAHAWP010000006	98.99
KH61	*Bacillus mexicanus* FSQ1^{T}	JAHAWP010000006	98.60
KH41	*Bacillus tequilensis* KCTC 13622^{T}	AYTO01000043	99.05

KH5	*Rhodococcus electrodiphilus* JC435^T	LT630357	98.93
KH53	*Gordonia amicalis* NBRC 100051^T	BANS01000072	98.80
KH37	*Gordonia amicalis* NBRC 100051^T	BANS01000072	98.34

Table S3. Crude oil degradation rates of bacterial isolates under varying NaCl concentrations.

No.	Strains	Estimated percentage degradation rate (%) of 1% (v/v) crude oil by bacterial isolates based on 2,6-DCPIP assay after 7 days, evaluated under varying NaCl concentrations				
		0%	1%	2%	3%	4%
1	*Stutzerimonas* sp. KH52	23.33 ± 2.52	24.67 ± 1.53	20.67 ± 2.52	17.33 ± 1.53	14.67 ± 1.53
2	*Pseudomonas* sp. KH55	25.67 ± 2.52	28.33 ± 2.52	25.33 ± 1.53	17.67 ± 2.52	15.33 ± 1.15
3	*Bacillus amyloliquefaciens* KHB35	26.50 ± 1.80	32.33 ± 2.52	34.67 ± 2.52	27.67 ± 2.52	22.67 ± 1.53
4	*Pseudomonas* sp. KH23	15.83 ± 0.29	18.33 ± 1.53	17.67 ± 2.52	15.67 ± 2.52	10.67 ± 1.53
5	*Pseudomonas aeruginosa* KH58	26.67 ± 2.52	24.33 ± 1.53	25.33 ± 1.53	19.67 ± 1.53	17.00 ± 1.00
6	*Pseudomonas aeruginosa* KH36	14.67 ± 1.53	16.67 ± 1.53	21.33 ± 2.52	21.67 ± 3.51	14.67 ± 2.52
7	*Pseudomonas* sp. KH51	16.67 ± 1.53	18.67 ± 2.52	19.67 ± 2.52	19.00 ± 1.73	11.67 ± 1.53
8	*Pseudomonas veronii* KHB29	31.00 ± 2.00	33.00 ± 2.65	32.33 ± 2.52	28.67 ± 2.08	20.67 ± 2.52
9	*Aeromonas* sp. KH75	30.67 ± 2.52	30.33 ± 1.53	28.33 ± 2.08	25.67 ± 2.08	16.67 ± 2.52

10	*Serratia* sp. KH11	20.00 ± 2.00	21.33 ± 1.53	23.33 ± 1.53	18.33 ± 1.15	16.00 ± 2.00
11	*Enterobacter* sp. KH77	15.67 ± 2.52	19.33 ± 2.52	21.67 ± 2.52	20.67 ± 2.52	17.67 ± 1.53
12	*Stenotrophomonas* sp. KH78	17.67 ± 2.52	17.33 ± 2.08	20.67 ± 3.51	22.67 ± 2.08	15.67 ± 2.52
13	*Stenotrophomonas* sp. KH46	14.67 ± 2.52	19.33 ± 2.08	22.00 ± 2.65	23.33 ± 1.53	17.33 ± 3.06
14	*Stenotrophomonas* sp. KH63	21.67 ± 2.52	22.67 ± 2.52	23.67 ± 2.52	19.67 ± 2.52	15.67 ± 1.53
15	*Stenotrophomonas* sp. KH12	21.00 ± 2.00	21.33 ± 2.08	19.67 ± 2.52	18.00 ± 2.00	14.67 ± 1.53
16	*Cupriavidus* sp. KH34	17.33 ± 1.15	18.33 ± 2.08	19.67 ± 2.52	19.00 ± 1.73	11.67 ± 1.53
17	*Achromobacter* sp. KH45	27.67 ± 2.52	28.67 ± 2.52	29.67 ± 2.52	23.67 ± 2.52	21.67 ± 1.53
18	*Achromobacter* sp. KH57	17.00 ± 2.00	25.33 ± 1.53	26.67 ± 2.08	17.00 ± 2.00	13.33 ± 1.53
19	*Achromobacter* sp. KH59	22.67 ± 1.53	22.67 ± 2.08	22.67 ± 1.15	16.33 ± 0.58	14.33 ± 1.53
20	*Achromobacter* sp. KH62	29.00 ± 2.00	32.00 ± 2.00	29.67 ± 2.31	29.67 ± 2.08	20.67 ± 2.52
21	*Niveispirillum* sp. KHB59	32.00 ± 2.00	34.33 ± 2.52	30.67 ± 2.52	25.67 ± 2.52	17.67 ± 1.53
22	*Brevundimonas* sp. KH74	22.33 ± 2.52	25.67 ± 2.08	27.67 ± 1.53	28.67 ± 1.53	20.67 ± 2.08
23	*Staphylococcus* sp. KH21	12.67 ± 2.52	17.67 ± 2.52	21.33 ± 2.52	22.67 ± 2.08	14.67 ± 2.52
24	*Staphylococcus* sp. KH33	11.67 ± 2.52	20.33 ± 1.53	22.67 ± 2.08	20.33 ± 2.08	15.67 ± 2.52
25	*Staphylococcus* sp. KH32	24.67 ± 2.52	24.33 ± 2.52	21.33 ± 2.52	19.33 ± 1.53	14.67 ± 2.52

26	*Pseudomonas tohonis* KH35	18.00 ± 1.00	18.67 ± 2.52	20.67 ± 2.52	17.67 ± 2.52	15.67 ± 1.53
27	*Bacillus velezensis* KH73	18.00 ± 2.00	22.67 ± 2.52	25.33 ± 2.52	19.67 ± 2.08	15.33 ± 1.53
28	*Bacillus* sp. KH42	21.67 ± 2.52	25.33 ± 2.52	22.33 ± 2.08	19.33 ± 2.08	18.67 ± 2.52
29	*Bacillus* sp. KH72	28.00 ± 1.00	30.00 ± 2.65	26.00 ± 1.73	24.67 ± 2.52	21.33 ± 1.15
30	*Bacillus mexicanus* KH44	25.33 ± 0.58	31.33 ± 1.15	30.67 ± 0.58	30.67 ± 0.58	25.67 ± 2.52
31	*Bacillus* sp. KH43	19.33 ± 2.52	20.33 ± 2.08	23.00 ± 2.65	19.67 ± 2.31	15.67 ± 2.08
32	*Bacillus tequilensis* KH61	18.67 ± 2.52	21.00 ± 1.73	20.67 ± 3.51	18.33 ± 2.52	16.33 ± 1.53
33	*Bacillus* sp. KH41	21.00 ± 2.08	21.33 ± 2.08	22.33 ± 2.08	20.67 ± 1.53	17.00 ± 2.00
34	*Rhodococcus* sp. KH5	33.00 ± 2.00	35.00 ± 2.65	35.00 ± 2.65	31.33 ± 2.52	17.67 ± 2.52
35	*Gordonia amicalis* KH53	28.00 ± 3.00	36.00 ± 2.00	34.00 ± 2.65	29.00 ± 2.00	28.67 ± 2.08
36	*Gordonia* sp. KH37	26.00 ± 2.00	33.33 ± 2.31	33.00 ± 2.65	28.67 ± 1.53	28.00 ± 1.00
